# Establishment of clinical exercise physiology as a regulated healthcare profession in the UK: a progress report

**DOI:** 10.1136/bmjsem-2024-002033

**Published:** 2024-06-18

**Authors:** Helen Jones, Anthony Crozier, Keith George, Gemma Miller, Greg P Whyte, Joanna Rycroft, Andrew Scott, John P Buckley, Gordon McGregor, Christopher David Askew, Sandy Jack, Steffan Birkett, David R Broom, Keith Tolfrey, Anna Campbell, Dawn A Skelton, Lizanne Steenkamp, Jude Savage, Daniel J Green

**Affiliations:** 1 RISES, Liverpool John Moores University, Liverpool, UK; 2 Sport, Health and Exercise Science, University of Portsmouth, Portsmouth, UK; 3 School of Allied Health Professions, Keele University, Keele, UK; 4 Sport and Exercise Science, Coventry University, Coventry, UK; 5 University Hospitals Coventry and Warwickshire NHS Trust, Coventry, UK; 6 School of Health and Sport Sciences, University of the Sunshine Coast, Maroochydore DC, Queensland, Australia; 7 Sport and Exercise Science, University of Southampton, Southampton, UK; 8 Sport and Exercise Science, Manchester Metropolitan University, Manchester, UK; 9 School of Sport, Exercise and Health Sciences, University of Loughborough, Loughborough, UK; 10 Sport Exercise and Health, Edinburgh Napier University, Edinburgh, UK; 11 Research Centre for Health (ReaCH), Glasgow Caledonian University, Glasgow, UK; 12 The Academy for Healthcare Science, Leicester, UK; 13 Sport and Exercise Science, University of Western Australia, Perth, Western Australia, Australia

**Keywords:** Exercise rehabilitation, Physiology, Communicable disease, Exercise physiology

## Abstract

In 2021, a ‘call to action’ was published to highlight the need for professional regulation of clinical exercise physiologists to be established within UK healthcare systems to ensure patient safety and align training and regulation with other health professions. This manuscript provides a progress report on the actions that Clinical Exercise Physiology UK (CEP-UK) has undertaken over the past 4 years, during which time clinical exercise physiologists have implemented regulation and gained formal recognition as healthcare professionals in the UK. An overview of the consultation process involved in creating a regulated health profession, notably the development of policies and procedures for both individual registration and institutional master’s degree (MSc) accreditation is outlined. Additionally, the process for developing an industry-recognised scope of practice, a university MSc-level curriculum framework, the Academy for Healthcare Science Practitioner standards of proficiency and Continuing Professional Development opportunities is included. We outline the significant activities and milestones undertaken by CEP-UK and provide insight and clarity for other health professionals to understand the training and registration process for a clinical exercise physiologist in the UK. Finally, we include short, medium and long-term objectives for the future advocacy development of this workforce in the UK.

**WHAT IS ALREADY KNOWN ON THIS TOPIC**

- Clinical exercise physiologists are recognised in providing safe, effective, personalised and optimised exercise interventions for primary and secondary prevention across the spectrum of chronic diseases.

**WHAT THIS STUDY ADDS**

- As of December 2021, the Academy for Healthcare Science-registered clinical exercise physiologists are regulated healthcare professionals in the UK.
- An approved scope of practice exists for clinical exercise physiologists in the UK, alongside an accredited master’s degree-level curriculum framework for university courses.

**HOW THIS STUDY MIGHT AFFECT RESEARCH, PRACTICE OR POLICY**

- This study will help inform the potential development of MSc apprenticeships for clinical exercise physiologists and the redevelopment of future iterations of the CEP-UK scope of practice.

## Background

There is overwhelming evidence that regular exercise is vital for the successful prevention and management of long-term health conditions.^1–3^ Indeed, for many conditions, exercise therapy may be as effective as established pharmacological therapies.^3–5^ Exercise therapy is highly cost-effective which is increasingly recognised in the UK. For example, the 2019 National Health Service (NHS) ‘Long Term Plan’ advocated exercise programmes for patients with cardiovascular disease to prevent 14 000 premature deaths,^6^ and the Office for Healthcare and Disparities (formerly Public Health England) has acknowledged that embedding physical activity into clinical care pathways in acute settings was required.^7^ Notwithstanding this identified need, an audit of clinical exercise services in the UK conducted in 2020 suggested that service delivery was piecemeal and delivered by a diverse range of individuals with significant variability in training background and/or qualification(s). Indeed, some individuals operating in those roles were identified as having no formal training in clinical, human or exercise physiology.^8^ This variation was at least partially explained by the lack of any formal regulation of clinical exercise physiologists in the UK to ensure safe, effective, personalised and optimised exercise interventions for primary and secondary prevention across the spectrum of chronic diseases. Internationally (eg, Australia), tertiary qualified exercise professionals with formal registration and/or accreditation have been established and recognised as allied health professionals for ~20 years.^9 10^ These health professionals are identified as those qualified to screen and triage, complete functional assessments, prescribe and deliver safe and effective exercise, and to support behaviour change interventions in the prevention, treatment and management of long-term and complex medical conditions.^9–15^ These professionals hold the title of clinical exercise physiologists (or similar).^10 12 13 16 17^

In 2019, a Research England International Investment Initiative Grant was awarded to the ‘iCardio’ Project led by Liverpool John Moores University and the University of Western Australia (principal investigators coauthors Jones and Green). This 5-year programme grant had an initial focus to critically assess clinical exercise service delivery in the UK and then, using guidance from Australia as a best practice model, explore the possibility of a formal and regulated professional career structure for clinical exercise physiologists in the UK. The iCardio grant has provided a platform and ‘seed-corn’ funding for the agendas and activity described in detail as follows.

## Summary of call to action

As a direct result of the iCardio Project, a group of UK clinical exercise physiologists (at that time unregistered), academics researching and teaching clinical exercise physiology, representatives from relevant organisations (eg, vocational education providers), and international collaborators with knowledge pertaining to the establishment of clinical exercise physiology standards and accreditation processes, published a call to action to establish clinical exercise physiology as a recognised and regulated health profession in the UK.^11^ In the call-to-action statement, a roadmap was outlined, and an interim steering group convened. This represented the seminal stage of establishment of Clinical Exercise Physiology UK (CEP-UK), which established a mission to provide leadership and to develop formal registration and regulation of a ‘clinical exercise physiologist’ title in the UK (table 1). The overarching aims of the group included to define the clinical exercise physiologist scope of practice; to create a formal register for clinical exercise physiologists on a Professional Standards Authority (PSA)-accredited register; to develop a university curriculum that aligns with evidence-based accreditation standards; to collaborate and create professional development opportunities and endorsed educational pathways; and to promote university-trained registered clinical exercise physiologists in the public and private sector within the UK.

**Table 1:** Key actions identified in the 2021 call to action

Strategic change	Identified action	Outcome
Consultation	Approach other organisations on the Professional Standards Authority register for insight into the process and potential for combining resources.	Achieved—June 2020 an approach was made to the Registration Council for Clinical Physiologists (RCCP)*
Consultation	Explore how to engage with local and national government and NHS to advocate the clinical exercise physiologist role in the healthcare system including placements for training and accreditation purposes.	Ongoing
Professional standards	Draft the scope of practice and standards of proficiency for consultation.	Achieved—scope of practice was agreed May 2021. Standards of proficiency were agreed to use RCCP*
Professional standards	Engage other health professionals (eg, sport and exercise medicine consultants, GPs, physiotherapist, commissioners) on the draft	Achieved—for example, via curriculum sharing event September 2022 and individuals and organisations listed on the curriculum framework document
Regulatory body	Establish the regulator including register holder, accreditation body and professional development system and register the title.	Achieved—the RCCP* in August 2021 with the clinical exercise physiology register opening in December 2021 Achieved—adopted Continuing Professional Development process of RCCP*
Education and accreditation	Bring together universities to discuss potential degree course content and agree on an accreditation process and framework.	Achieved—consulted and drafted. Endorsed and published in May 2022
Establish leadership	Establish the ‘council’ that leads and potentially governs the regulatory body that takes over from the interim steering group.	CEP-UK was established in October 2021 and replaced the steering group. Ongoing development on the structure and wider inclusion through the engagement of current practitioners working as a clinical exercise physiologist in the UK

*The initial approach and subsequent progress outlined throughout this document until June 2021 was with the RCCP. In June 2021, the RCCP became a wholly owned subsidiary of the Academy for Healthcare Science (AHCS). The AHCS holds an accredited register with many parts that includes clinical physiologists. Two registers continued during the transfer of business.

CEP-UK, Clinical Exercise Physiology UK; GPs, general practitioners; NHS, National Health Service.

The aims of this progress report are to (1) describe the process CEP-UK employed to establish and regulate clinical exercise physiologists, (2) outline the current recognition and advocacy in the UK regarding the registration of clinical exercise physiologists, and (3) outline the areas of identified focus and future action in the short (up to 1 year), medium (1–3 years) and long term (3 years plus).

## Equity, diversity and inclusion statement

The author group is gender balanced and consists of junior, mid-career and senior researchers from sport and exercise sciences backgrounds. Members of the group come from three different UK and international countries with varying demographic and cultural differences.

## The process employed to establish and regulate clinical exercise physiologists

### Establish an appropriate regulator for healthcare professionals

In the UK, the PSA is an independent organisation, accountable to UK Parliament, tasked with reviewing the work of the regulators of health and care professionals, governed by legislation. The PSA also accredits organisations that register health and care practitioners not regulated by legislation, establishing accredited registers. PSA-accredited registers demonstrate that a registered health professional who is not regulated by law has achieved the highest standards in governance, education and training and professional standards required within their scope of practice, while also demonstrating competency in the management of the register, complaints handling and information including fitness to practice monitoring. The focus of regulation in this manner is ultimately to ensure patient safety.

The Registration Council for Clinical Physiologists (RCCP*) held a PSA-accredited register for clinical physiologists (specialisms include cardiac, gastrointestinal, respiratory, sleep and neurophysiologists, audiologists, educational audiologists and hearing therapists) until the transfer to the AHCS. Clinical exercise physiologists were identified as having similarities to clinical physiologists in terms of knowledge, skills and training, and the RCCP had the ability and processes in place to accredit university-level degree courses. In June 2020, CEP-UK approached and subsequently agreed to work with the RCCP to establish clinical exercise physiologists on their PSA-accredited register.

### Scope of practice and standards of proficiency

A scope of practice was developed by CEP-UK and benchmarked against international standards (scope of practice v2[2].pdf (clinicalexercisephysiology.org.uk). A scope of practice describes the procedures, actions and processes that a healthcare practitioner is permitted to undertake, in keeping with the terms of their regulator.^18^ It is required for all regulated healthcare professionals in the UK.^19^ A unique aspect of the clinical exercise physiologist scope of practice was that it contained a list of long-term health conditions (cancer, cardiovascular, frailty, renal, mental health, metabolic, musculoskeletal, neurological/neuromuscular and respiratory/pulmonary) for which there is an evidence base supporting the efficacy of clinical exercise services.^3^

Similarly to the scope of practice, all regulated healthcare professionals in the UK work towards a specific standards of proficiency (SoP) document which covers professional autonomy and accountability, and the skills required for practice. In addition, the AHCS requires all registrants to comply with the AHCS Good Scientific Practice (GSP) standards. After consideration by CEP-UK, this was also deemed applicable for clinical exercise physiologists.

### Curriculum standards

While regulation was being pursued, it was recognised that curriculum standards were required to provide guidance for new university programmes on what would be required to meet accreditation standards. The curriculum standards were developed for creating new clinical exercise physiology master’s degrees (MSc). Curriculum standards ensure consistency in knowledge, skills and experience of the workforce, and help university programme teams to interpret definitions in the scope of practice.

The curriculum framework developed by CEP-UK underwent several iterations (members listed on the published document), was benchmarked against international standards for clinical exercise physiologists (or equivalent) and was comparable with UK standards for other healthcare professionals.^20^ The published curriculum framework consists of six broad headings, including (1) pathophysiology and clinical management, (2) screening and risk stratification, (3) assessment of health status and functional capacity, (4) design of exercise interventions, (5) exercise implementation and delivery, and (6) behaviour change and communication. Each broad standard outlines the specific knowledge and skills required in more detail. The curriculum framework also outlines the compulsory practice-based learning requirements and practical competency assessments.

**Figure 1:**
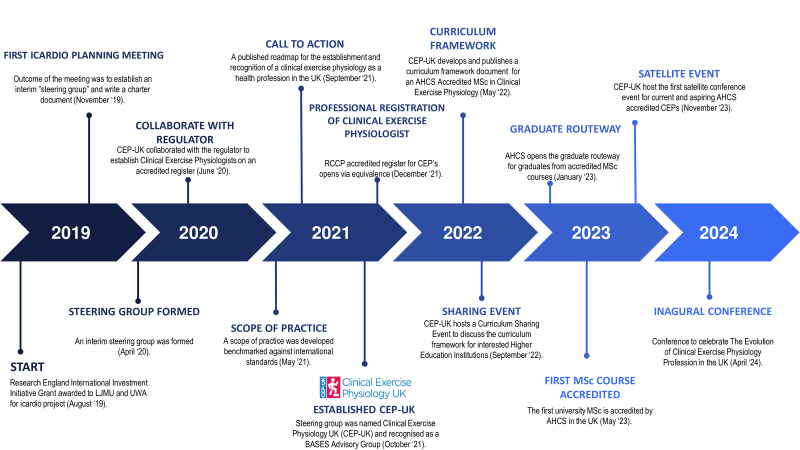
CEP-UK milestone timeline. AHCS, Academy for Healthcare Science; BASES, British Association of Sport and Exercise Sciences; CEP-UK, Clinical Exercise Physiology UK; MSc, master’s degree; RCCP, Registration Council for Clinical Physiologists.

CEP-UK sought consultation and feedback with academics and practitioners in the field via an online survey before publishing the curriculum framework. Respondents (n=31) consisted of academics (65%), clinical exercise practitioners (19%), clinical exercise service leads (7%) and other professional organisations (9%). Around 90% of respondents acknowledged that the curriculum framework content devised by CEP-UK met the standards required of an MSc-level degree, with 94% identifying sufficient detail within the document to allow universities to design and implement content within their postgraduate programmes. After acting on relevant feedback, the final iteration of the curriculum framework was presented at a curriculum sharing event to disseminate this document to stakeholders with 80 attendees representing >20 institutions from the UK. The curriculum framework document was published on the CEP-UK website in May 2022 (figure 1). This document will be reviewed and updated after 3 years (May 2025).

### Registration

#### Equivalence pathway to registration

**Figure 2:**
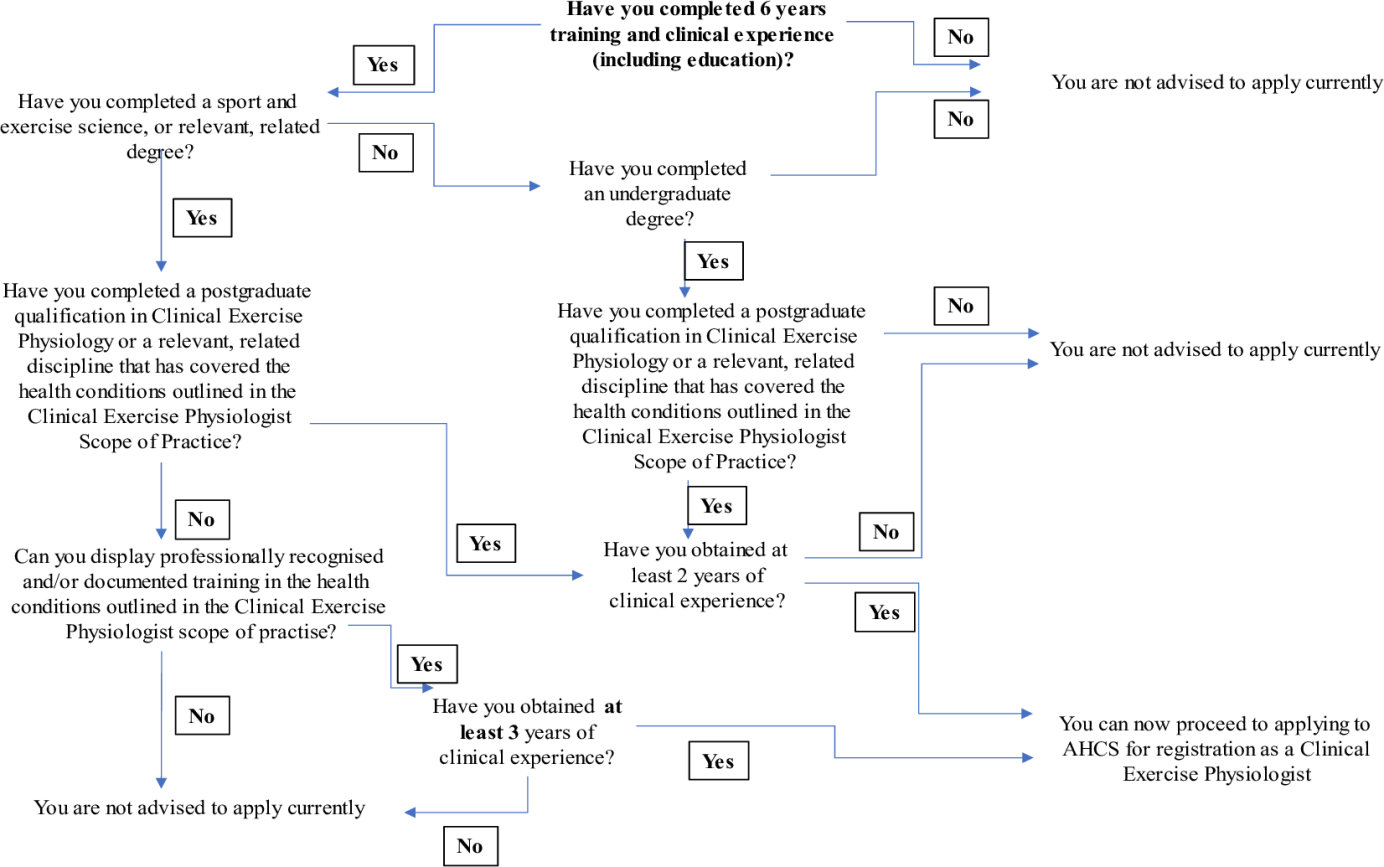
Equivalence pathway for individual registration. AHCS, Academy for Healthcare Science.

Prior to registration, unregulated clinical exercise physiologists have been working across public and private sectors within clinical exercise services (~890) in the UK for over 25 years.^8 21^ These individuals identified themselves by various titles (eg, exercise physiologists, exercise scientists, sport and exercise scientists, exercise specialists and advanced exercise instructors) and held a variety of qualifications.^8 21^ It was apparent from the previous audit of clinical exercise services^8^ that many of these individuals possessed associated undergraduate degrees (eg, sport and exercise sciences) and postgraduate degrees (eg, exercise science) and/or vocational qualifications (eg, advanced exercise instructor in cardiac rehabilitation), together with years of hands-on practical experience that would likely meet the required standards to become a registered clinical exercise physiologist (figure 2). It was therefore necessary to develop a pathway for these individuals to become registered.

CEP-UK used the content within the curriculum framework and scope of practice to create a template application form to allow these professionals to display their knowledge, skills, behaviours and experience. The regulator (at that time the RCCP) submitted a change notification form to the PSA requesting the addition of clinical exercise physiology to their PSA-accredited register. In August 2021, the PSA accepted the addition of clinical exercise physiologists and the clinical exercise physiology equivalence pathway was opened in December 2021. This was effectively the time point at which clinical exercise physiologists became recognised and regulated health professionals in the UK. For an applicant to be successfully registered via the equivalence pathway, they need to have completed 6 years of training/work experience across education or clinical exercise settings. One option to fulfil this criterion is an undergraduate degree in a sport and exercise science-related subject, plus 3 years of experience and training in the delivery of clinical exercise covering all the pathologies outlined in the CEP-UK scope of practice. A second option is an undergraduate degree, supplemented by a postgraduate degree in clinical exercise physiology or equivalent, and then 2 years of experience and training in the delivery of clinical exercise. In both options, applicants must display knowledge and experience in all pathologies within the CEP-UK scope of practice. Qualifications are required to support applications, alongside current employer references, work history and an overview of an applicant’s knowledge, skills and work experience across each of the CEP-UK curriculum standards. For each pathology/long-term condition, applicants need to demonstrate their competency in: pathophysiology, screening and risk assessment, designing an exercise intervention, exercise delivery and behaviour change and communication. Additionally, an applicant is required to provide evidence of professional indemnity insurance and public liability insurance to access the register (either via employer or personal indemnity insurance).

#### Scrutineer training and application assessment

A requirement for the accredited register was to appoint application scrutineers for the assessment of equivalence applications. Scrutineers were selected from the CEP-UK steering group based on their ability to become registered clinical exercise physiologists themselves through the equivalence pathway process and satisfactory compliance to the scrutineer role description. Scrutineers undertook an onboarding process whereby they underwent training in application assessment, including group workshops, consensus agreement regarding application merit and quality assurance checks through the process of auditing all assessments during the initial phase of equivalence. In-depth training was given concerning the criteria for acceptance for applicants, including qualifications, experience and coverage of core competencies, alongside application/document completion and potential outcomes of any application (accept, request further information, reject or assessor review for additional assessment). The target for assessment completion is 28 days from receipt of any application.

#### Graduate route to registration through AHCS (as part of the transfer of the RCCP to the AHCS) accreditation from January 2023

**Figure 3:**
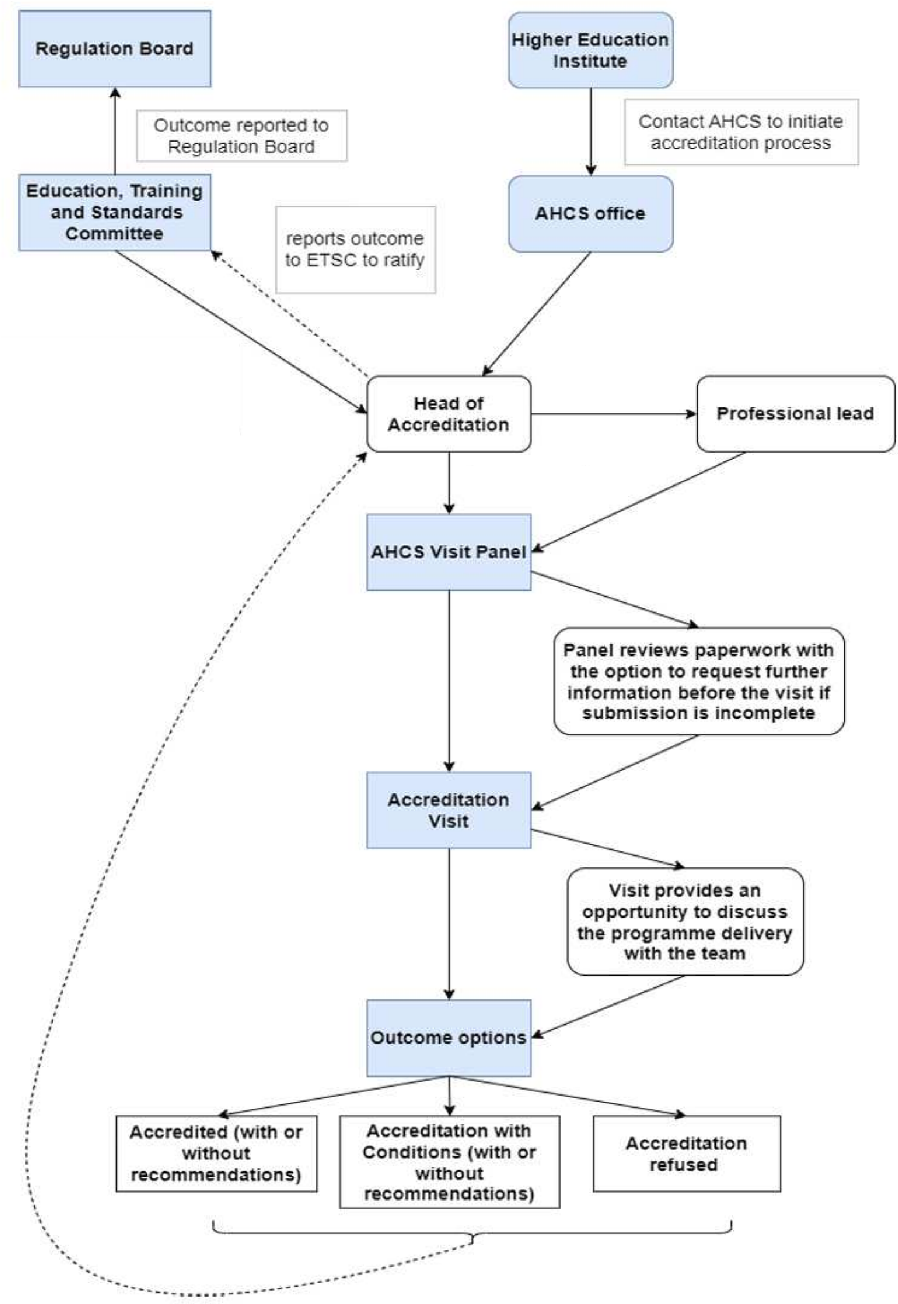
AHCS accreditation process flow chart. AHCS, Academy for Healthcare Science.

The CEP-UK curriculum framework and scope of practice alongside the AHCS Practitioner SoP and AHCS GSP standards are collectively used by the AHCS to assess MSc courses for accreditation. CEP-UK held a curriculum sharing event in September 2022 to help university programme teams understand the requirements for accreditation, share ideas on how to deliver a clinical exercise physiology MSc programme as well as meeting clinical and practical requirements. The AHCS opened the graduate route in January 2023 for MSc programmes to apply to be assessed for accreditation by the AHCS (figure 3). The accreditation involves a desktop review of submitted paperwork by the AHCS head of accreditation and a registered clinical exercise physiologist with expertise and experience of higher education processes. If the desktop review is deemed satisfactory, then a site visit is arranged with the university programme team, local management as well as placement providers. Feedback is provided at all stages of the process. Recommendations to the Education, Training and Standards Committee (ETSC) following the site visit include accreditation, accreditation secondary to satisfactorily addressing conditions or accreditation refused. Once the university MSc course has final notification of accreditation in writing, their students graduating from the MSc course are automatically eligible for registration as a clinical exercise physiologist on the AHCS-accredited register.

The graduate route for registration involves the completion of an accredited postgraduate MSc in clinical exercise physiology after an undergraduate degree involving the cognate discipline knowledge. Graduates are required to pass each module within the MSc course, including the fulfilment of a minimum 250 hours of practice-based learning, a minimum 140 hours of which is required within clinical placement and successful completion of the Clinical Assessment of Competencies (CAP) examinations for each set of curriculum standards. Completion and passing of an AHCS-accredited MSc, alongside the CEP-CAP assessment, will ensure that graduates have met the CEP-UK scope of practice and AHCS Practitioner SoP and GSP, and will therefore be eligible for registration. The first MSc Clinical Exercise Physiology Programme was accredited in August 2023.

### Maintenance of registration as a clinical exercise physiologist

A clinical exercise physiologist is registered (via the equivalence pathway or graduate route) for a period of 1 year. Following this time period, individuals are required to reconfirm registration on an annual basis and pay the required fees.

A requirement for ongoing registration is Continuing Professional Development (CPD). To minimise the risk of harm to patients, AHCS registrants are required to demonstrate that they are fit to practise through CPD, both in terms of their competency and conduct. This is important in supporting a culture of continuous learning and improvement, as well as ensuring the professionalism of registrants through shared experience and community development. CPD helps registrants maintain or develop competence and fitness to practise and is an essential requirement for registration. CPD can take many forms, such as professional activities, formal education, work-based learning or independent learning. At present, individuals are required to complete sufficient self-managed CPD per annum that demonstrates currency of learning within their specialism, while keeping a log of all activities ready for AHCS audit requirements. If audited, registrants submit a personal statement summarising practice history for the previous 2 years and any CPD activities undertaken. If they do not maintain CPD, individuals are removed from the AHCS register.

## Clinical exercise physiologists currently registered

As of May 2024, there are 94 registered clinical exercise physiologists in the UK with 1 further application in process. In addition, there are 238 applications that have been started on the AHCS system but not yet submitted. There are two accredited clinical exercise physiology MSc courses (one with conditions) with three undergoing accreditation assessment. There are a further 11 universities that have registered an interest in developing an accredited curriculum for assessment and are at various stages of the process in development. A conservative projection for the workforce using equivalence and graduate pathways is ~1678 registered clinical exercise physiologists by 2029.

### Professional body for clinical exercise physiologists

The British Association of Sport and Exercise Sciences (BASES) is the professional body for sport and exercise sciences in the UK with a mission to drive excellence in sport and exercise sciences through the promotion of evidence-based practice and the development and enhancement of professional and ethical standards. The similarity of aims and objectives between CEP-UK and BASES allowed an agreement to be signed in October 2021 between both parties with the understanding that BASES would house CEP-UK within their organisational structure (Divisions | BASES). CEP-UK is recognised as a BASES advisory group that retains part of the BASES Articles of Association, with mutually agreed terms of reference, and internal reporting directly to the BASES Board. The BASES organisation provides administrative and legal support to CEP-UK as part of the partnership, in addition to a shared commitment to develop advocacy, CPD provision for members, alongside access to discounted professional indemnity insurance.

### Advocacy for clinical exercise physiologists

**Figure 4:**
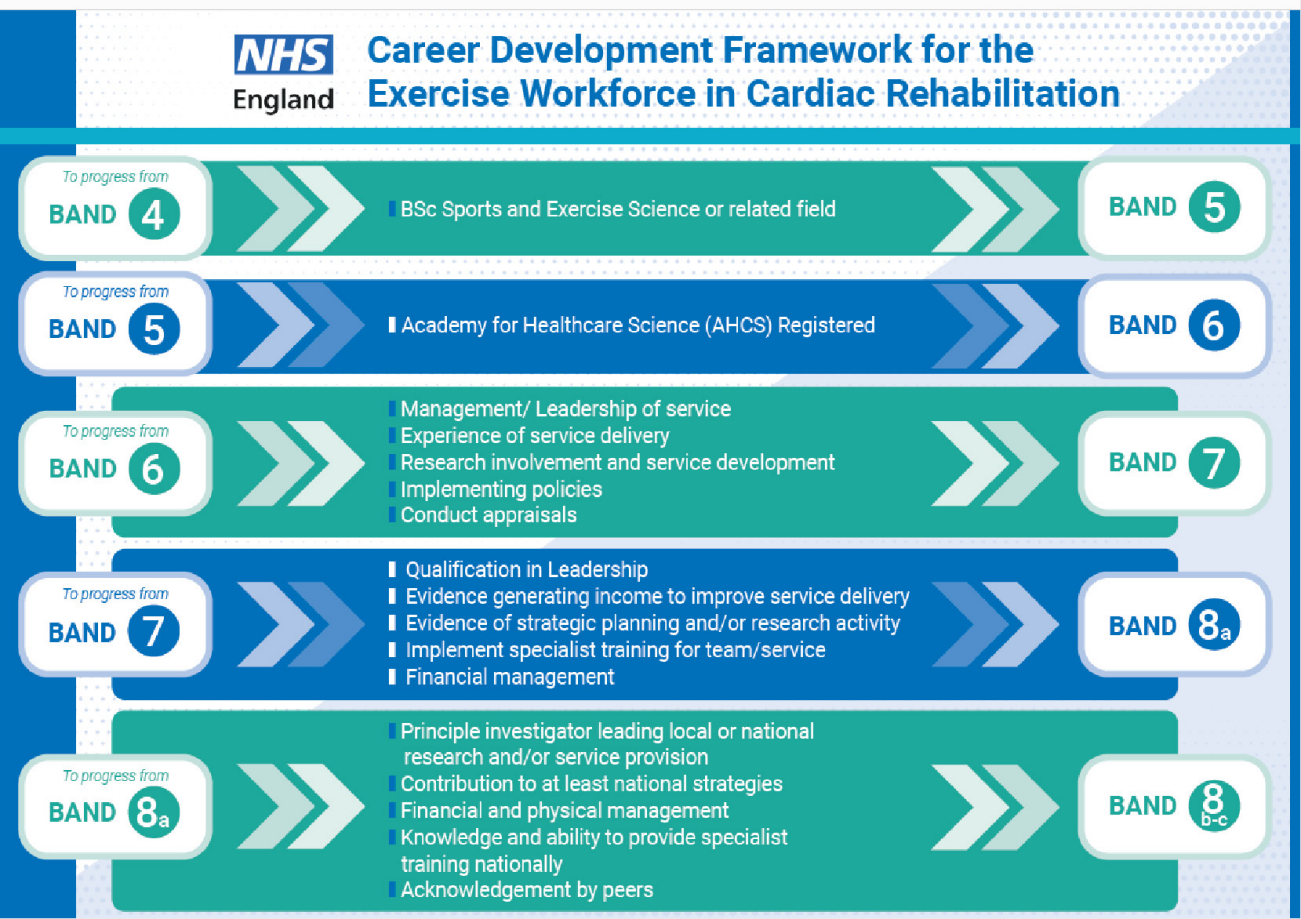
Career development framework for the exercise workforce in cardiac rehabilitation NHS England (HEE legacy) Star bid. BSc, Bachelor of Science; HEE, Health Education England; NHS, National Health Service.

Given that formal registration and regulation of the clinical exercise physiology title in the UK, the initial mission of CEP-UK, has been achieved, CEP-UK vision has moved to setting standards, co-creating CPD opportunities and advocating for AHCS-registered UK clinical exercise physiologists. Our vision is that registration and regulation of clinical exercise physiologists would provide equal standing with other registered health professions regarding competency levels leading to increased respect among with health professional peers in the future.^21 22^ Realisation of this aim has begun and is evidenced in the following ways:

- Job descriptions and person specifications for new jobs advertised in the NHS and private healthcare include AHCS registration or working towards AHCS registration as desirable and essential criteria, respectively, for clinical exercise services for different long-term conditions (eg, cardiac, pulmonary, cancer).
- NHS England commissioned, via a Star award, job description and person specification standard templates along with a career development framework for an exercise workforce in cardiac rehabilitation from band 4 associate exercise practitioner, through to band 8a–8c consultant clinical exercise physiologist (figure 4). These documents are in the public domain (HEE Star: Accelerating workforce redesign | Health Education England) and have been circulated to all national cardiac network leads via an NHS England and British Heart Foundation Cardiac Rehabilitation Sharing and Learning Workshop.
- CEP-UK has been working with NHS Careers and Futures, leading to the profession of clinical exercise physiologist being outlined on their respective websites under physiological healthcare science specialisms since September 2023 (Clinical exercise physiologist | Health Careers) akin to other health professions, such as physiotherapist.
- Members of CEP-UK have presented at meetings and conferences on regulation and registration of clinical exercise physiologists and the role of a clinical exercise physiologist (eg, London Respiratory Clinical Network NHS England, physiotherapist oncology network, cardiac rehabilitation network, Macmillan allied health professional advisory network, BASES webinar series) and CEP-UK has been recognised as an important body to contribute to other groups (eg, Centre for Perioperative Care, NHS Long Term Workforce Planning Group, Chartered Society for Physiotherapists’ Collaborate Don’t Compete Project steering group, Pulmonary Rehabilitation Workforce group).
- Research studies have been conducted and published in academic journals, examining the role of a clinical exercise physiologist in clinical exercise services in the UK. These are real-world examples of successful clinical exercise physiologists working within the NHS and the third sector, with peer recognition regarding their knowledge and skills as an integral part of the workforce.^21 22^

Such exposure has increased and will continue to increase awareness of clinical exercise physiologists within the UK healthcare system including the NHS, creating opportunities for new graduates from accredited MSc courses (eg, AHCS-registered clinical exercise physiologists via the graduate route) and career development for employed AHCS-registered clinical exercise physiologists to progress their careers in management, including the potential of setting up new services. At the same time, promoting the career as a clinical exercise physiologist to current and potential undergraduate students.

### Future directions

The ongoing work for CEP-UK includes the implementation of short, medium and long-term objectives (summarised in table 2). In the short term, the focus remains on raising the awareness of the regulation and accreditation processes that clinical exercise physiologists are now undertaking to become formally acknowledged as healthcare professionals. Conferences and events that develop a shared community of practice will enable advocacy and reach across a variety of stakeholders (eg, clinical exercise physiologists, healthcare professionals, universities) with ongoing evaluations of the current structure ensuring relevance within the sector (inclusion of more practitioners) and sufficient support moving forward. A key medium-term objective is the development of the clinical exercise physiologist workforce through various training and education pathways (eg, apprenticeships), with emphasis on increased workplace integration and learning akin to other healthcare professions. Longer term, CEP-UK envisages the development of new clinical exercise services with clinical exercise physiologists as integral part of all multidisciplinary healthcare teams and at the forefront of service delivery. Such long-term objectives would allow alignment with international clinical exercise physiologists or equivalent peers (eg, Australia) as well as alongside healthcare professionals within the clinical exercise service space in the UK.

**Table 2:** CEP-UK future aims and objectives

Action	Potential challenge
Short-term developments	
Inaugural CEP-UK conference April 2024	Embedding this as a regular event (biannual)
Progression in governance and oversight	Ensuring a fit for purpose structure is implemented and facilitates the growth of clinical exercise physiologists (CEPs) as a workforce in the UK
Communication strategy	Increasing engagement across CEPs and healthcare professionals
Medium-term	
Devising an apprenticeship framework	Whether universities are open to apprenticeships as an option for CEPs and whether they can get local agreement with NHS trusts or private health
Reviewing the MSc CEP curriculum framework	Timeline of reviewing in 2025 might be too early given the number of universities undergoing accreditation currently
Lobbying for CEPs to be listed as an exercise workforce in revised or new policy documents	Timelines of new or updated guidelines and ensuring CEPs are part of working groups
Devising a financial model for CEP-UK to be financially self-sustainable	Income from memberships and CPD
Longer-term	
Supporting the set-up of new services (eg, for multimorbid services, links with private sector)	Funding
Innovation to set up new CEP services (eg, primary care, private business)	
Long-term audit and return on investment evaluation (akin to Australia)	
Exploration of tariffs for student placements (akin to other UK health profession placements)	

CEP-UK, Clinical Exercise Physiology UK; CPD, Continuing Professional Development; MSc, master’s degree; NHS, National Health Service.

## Conclusion

While a platform has been developed and implemented by aspirational, ambitious and hard working pioneers in the pursuit of creating a regulated profession for clinical exercise physiology in the UK, the ultimate success of this endeavour, which is based on promoting patient and public safety, will depend on engagement of the practitioners in their own interests, and collaborations with other practitioner groups to cooperate and enhance effective healthcare for the UK. The initial mission of clinical exercise physiologist registration on a PSA-accredited register has been achieved and is a stepping stone to a larger vision whereby clinical exercise physiologists are acknowledged as a key component at the forefront of exercise delivery within clinical exercise services, both publicly and privately in the UK.
